# Fracture Resistance in Fibre-Reinforced Resin Composite Restorations in Deciduous and Permanent Molars: An Ex Vivo Study

**DOI:** 10.1016/j.sdentj.2024.06.017

**Published:** 2024-06-12

**Authors:** Hassan Mossad Hassan Negm, Dina Yousry Elkharadly, Sherine Badawy, Rania Rashad Omar Omar Taha

**Affiliations:** a Department of Conservative Dentistry, Faculty of Dentistry, October 6 University, Egypt; b Department of Pedodontics, Faculty of Dentistry, October 6 University, Egypt; c Department of Pediatric Dentistry, University of Science and Technology of Fujairah, UAE

## Abstract

**Aim:**

The aim of this study was to compare the fracture resistance levels of restored deciduous teeth and permanent molars restored with different materials, including ultra-polyethylene fibre tape (Ribbond-Ultra), fibre-reinforced resin composite EverX posterior, fibre-reinforced flowable resin composite EverX Flow and bulk-fill flow restorative material (Tetric N-flow) in the posterior region.

**Methods:**

We tested sixty-four caries-free human mandibular molars (32 deciduous molars and 32 permanent molars). Deep and wide Class I cavities were prepared in each tooth. The teeth were embedded in poly(methyl methacrylate) resin up to the cementoenamel junction, leaving the crown completely exposed. Oral temperature fluctuations were then simulated using two thousand thermocycling cycles, each lasting 30 seconds in the hot phase (approximately 35 °C). The loading rate for our force-fracture tests was set at 0.5 mm/min. Direct restorations were made using a spherical tip and applying the force perpendicular to the sample surface. Visual inspection of the fractured specimens, in combination with adhesive scanning electron microscopy (SEM) and finite element analysis (FEA), provided detailed insights into the failure modes and stress distribution at the restoration–tooth interface.

**Results:**

Teeth restored with fibre-reinforced composite (EverX Posterior) had the highest fracture resistance, followed by fibre-reinforced flowable resin composite (EverX Flow). Teeth restored with the ultra- polyethylene fibre tape (Ribbond-Ultra), followed by the flowable bulk-fill composite (Tetric N-flow) had the lowest resistance. Statistical analysis revealed significant differences between the groups, except for EverX Posterior and EverX Flow. A predictive algorithm was also proposed for the likelihood of restoration failure.

**Conclusion:**

Modern fibre-reinforced resin composites, such as EverX Posterior, effectively reinforce teeth against fractures, with success in both restored deciduous and permanent molars. Meanwhile, the use of polyethylene fibre tapes is less effective, and they involve a  time-consuming application.

## 1 Introduction

In restorative adhesive dentistry, the material selection and restoration design have been guided by clinical experience, manufacturer recommendations and trial-and-error, rather than the use of a rigorous quantitative approach, though such an approach may serve to optimise the results of the benchmark dental restorations. The challenges of using composite resins include issues such as inadequate conversion in deeper areas, increased polymerisation shrinkage and internal stresses, curing depth limitations, potential heat generation near the pulp, material handling and adaptation requirements, wear resistance, aesthetic considerations and ensuring effective adhesion to dentine and enamel (Baroudi and Rodrigues, 2015, Chisini et al., 2018, Demarco et al., 2023). Further challenges also include maintaining long-term stability and marginal integrity; managing post-operative sensitivity, sensitivity to clinical application techniques and thermal expansion; ensuring the longevity of the restoration; optimising handling properties; meeting patient acceptance criteria and considering cost effectiveness (Chisini et al., 2018).

Restoring teeth is about restoring functionality and protecting the normal, viable tooth structure, which is severely affected by the stresses following a fracture to an existing restoration (de Abreu et al., 2023, De Oliveira et al., 2020, Haridy et al., 2022). The use of composite fillings in the posterior region to ensure versatility and aesthetics has long focused on excellent bond strength, technically sensitive application and susceptibility to shrinkage and microleakage, since considering such factors may increase the longevity of the restoration (Rosatto et al., 2015). However, although each restoration material has specific properties, such as elasticity, hardness, fracture toughness, the coefficients of thermal expansion and shrinkage during curing, dentists typically specify the type of tooth—primary or permanent—regardless of morphology and without regard to the likelihood of fracture (Al-Nahedh and Alawami, 2020, Martins et al., 2020).

Better fracture resistance is offered by bulk-fill composite resins, which can be applied in thicker layers and polymerised in a single step (Papadogiannis et al., 2015). Incorporating nanofillers or polyethylene fibres further enhances resin composite strength. For example, Ribbond demonstrates high fatigue resistance, an elastic modulus comparable to dentine and efficient force transfer while also offering stress distribution and energy absorption properties (Foek et al., 2013). Fibre-reinforced resin composite restorations also enhance material structural properties by acting as effective crack stoppers (Foek et al., 2009, Garlapati et al., 2017, Gurbuz et al., 2008). For instance, EverX Posterior, a premixed composite containing numerous short E-glass fibres firmly integrated into a nanohybrid composite resin matrix, offers superior wear resistance and fracture toughness comparable to dentine (Goracci et al., 2014, Özyürek et al., 2018).

In this study, we compared four dental restorative resins [an ultra-polyethylene fibre tape (Ribbond-Ultra), fibre-reinforced resin composite (EverX Posterior), fibre-reinforced flowable resin composite (EverX Flow), and flowable bulk-fill composite (Tetric N-flow)] in applications to deciduous and permanent molars, to assess their fracture resistance in respect to minimising potential confounders. In a computational venture, we also sought to propose a mathematical algorithm for predicting the likelihood of restoration failure.

## 2 Materials and Methods

The materials utilised in this study encompassed a range of products designed for restorative dental procedures, each with distinct functions and compositions. Among them were poly-ethylene fibre (Ribbond-Ultra), known for its ultra-high strength and biocompatibility; a fibre-reinforced resin composite (EverX Posterior), incorporating E-glass fibres and barium borosilicate glass filler for increased strength; a fibre-reinforced flowable resin composite (EverX Flow), offering short fibre reinforcement for dentin replacement; and a bulk-fill flowable resin composite (Tetric N-flow), featuring a monomer matrix with inorganic fillers for light-cured bulk fill applications.

In total, 64 caries-free human mandibular molars (32 deciduous and 32 permanent molars). Deep and wide class I cavities were prepared in each tooth (eight per group), standardised to dimensions of 4 mm width and 4 mm depth, and restored with the designated restorative materials according to the manufacturer’s instructions. The exclusion criteria included molars with an unusual morphology in the occlusal surface, but with a largely consistent shape and size to minimise the possible confounders.

Each prepared tooth was washed with air–water spray, dried and etched with 37% orthophosphoric acid for enamel and dentine analysis. This was followed by priming and bonding procedures using OptiBond Extra Universal, a two-component adhesive, applied with a microbrush, and light curing for 10 s. The adhesive used was OptiBondExtra Universal, a two-component, light-cured adhesive, and the enamel and dentine etchant employed was Meta Biomed acid etching gel containing phosphoric acid and xanthan gum (Toz et al., 2015) (Supplementary Table 1). In both primary and permanent dentitions, Group I encompassed cavities restored using Ribbond-Ultra and Tetric N Flow bulk-fill composites. In Group II, EverX Posterior fibre-reinforced bulk fill resin composite was applied directly into the cavity and cured. Group III was treated with EverX Flow fibre-reinforced bulk fill flowable resin composite, while Group IV received Tetric N Flow bulk fill composite restoration.

The teeth were partially embedded in poly(methyl methacrylate) resin up to the cementoenamel junction, leaving the crown fully exposed. Diamond finishing burs and polishing discs were used to create a smooth, glossy surface. After restoration, the teeth were stored in distilled water at 37°C for 24h to simulate oral conditions. Thermocycling was conducted with 2000 cycles, each lasting 30 s in the hot phase (approximately 35°C) to simulate oral temperature fluctuations equivalent to six months of oral exposure. The loading speed for load-to-fracture tests was set at 0.5 mm/min. A spherical tip was used for direct restorations, and the force was applied perpendicular to the specimen surface. Supplementary Table 3 shows the PICOT attributes.

Visual inspection of fractured samples by light microscopy at a 50× magnification, combined with adhesive scanning electron microscopy (SEM) and finite element analysis (FEA), provided detailed insights into failure modes and stress distribution at the restoration-tooth interface. SEM examination involved mounting samples on conductive carbon tape, sputter-coating with gold, and examining fracture surfaces at various magnifications for a detailed microstructural analysis. FEA was conducted using ANSYS software to simulate the mechanical behaviour of the tooth-restoration complex under occlusal loading. A digital model was created that incorporated different adhesive layers and material properties to reflect actual conditions.

Mechanical loads were applied to simulate occlusal forces, and stress and strain distributions at the fracture interfaces were analysed. The mesh generation was carefully refined around critical areas to ensure accuracy, and boundary conditions were set to mimic realistic constraints. The failure patterns were then categorised based on the side of the fracture and stress concentration points, correlating the FEA predictions with the observed fracture patterns from light microscopy and SEM. This comprehensive approach allowed us to gain detailed insights into the stress distributions and failure mechanisms of the dental restorations, based on which we offer recommendations for improved material selection and bonding techniques.

A predictive algorithm was proposed based on geometric factors (composite thickness, tooth width, and initial crack length), in-vitro or ex-vivo environmental settings (heat flux, thermal conductivity, area, material constant and resin application time), and failure parameters (wear coefficient, normal/compressive load, sliding distance, temperature change, stress intensity factor range and crack measurements). The following pseudocode summarises the algorithmic logic:

*# Input for Material and tooth properties*

FUNCTIONS

getMaterialProperties (selectedMaterial)

getToothProperties (selectedDentition)

getStressConcentrationFactor (selectedDentition, toothWidth, compositeThickness)

getAdjustedForce (baseForce, selectedDentition)

getInterfaceQuality (selectedMaterial, selectedDentition)

getThermalStress (ΔT, selectedMaterial, selectedDentition)

getResidualStress (selectedMaterial)

FUNCTION predictFatigueCrack (ΔK, selectedMaterial, N)

FUNCTION predictFractureProbability (σ, σ_crit, selectedMaterial)

FUNCTION adjustToughnessForHydration (K_IC, time)

*# Main Prediction Function*

FUNCTION predictFracture ()

*# Retrieve input values*

Show RESULTS

## 3 Results

Here, we compared the maximum compressive loads of restored deciduous and permanent molars across four groups after the restorations. For deciduous molars, Group I demonstrated a mean maximum compressive load (149.91 ± 27.06), whereas Group II displayed a higher value (165.94 ± 25.83). Conversely, permanent molars in Group III exhibited a mean maximum compressive load (163.73 ± 28.53), while those in Group IV showed a slightly lower value of 153.21 ± 53.85. In the case of permanent molars, Group I displayed an average maximum compressive load (175.52 ± 31.67), whereas Group II exhibited a slightly elevated value (184.32 ± 21.24). Conversely, permanent molars belonging to Group III had a mean maximum compressive load of 151.64 ± 23.36, while those in Group IV showed a marginally lower values (149.39 ± 63.11).

Statistical analysis using ANOVA revealed a significant difference among the studied groups, with an F-value of 956.28 (*P* < 0.001). The comparison of the maximum compressive load among restored deciduous and permanent molars revealed significant differences across the groups. Deciduous molars in Group I exhibited a mean difference of 98.8 compared to Group II, indicating a substantially lower maximum compressive load capacity. Similarly, when comparing deciduous molars in Group I to permanent molars in Groups III and IV, mean differences of 86.12 and 43.87 were observed, respectively, highlighting the superior load-bearing capacity of permanent molars. Among the restored deciduous molars, those in Group II displayed a mean difference of 32.68 compared to permanent molars in Group III, showing a slight advantage in maximum compressive load. Nevertheless, the comparison between deciduous molars in Group II and permanent molars in Group IV revealed a significant mean difference of 54.93, indicating a notable difference in load-bearing capacity favouring the permanent molars. The permanent molars in Group III also showed a mean difference of 42.25 compared to permanent molars in Group IV, suggesting variations in load resistance even among restored permanent dentition. These findings underscore the importance of considering tooth type and developmental stage when evaluating load-bearing capabilities in dental restorations. The results of a post hoc Tukey’s test are shown in Table 1. All other measures failed to produce statistically significant differences.

**Table 1:** Maximum Compressive Load Among Deciduous and Permanent Molars.

Comparison	Mean Difference	95 % CI	p-Value
Deciduous Molars (Group I) vs Deciduous Molars (Group II)	98.8	962.29––855.32	<0.001
Deciduous Molars (Group I) vs Permanent Molars (Group III)	86.12	929.60––822.64	<0.001
Deciduous Molars (Group I) vs Permanent Molars (Group IV)	43.87	47.35––350.39	<0.001
Deciduous Molars (Group II) vs Permanent Molars (Group III)	32.68	20.80–––86.17	0. 0316
Deciduous Molars (Group II) vs Permanent Molars (Group IV)	54.93	51.45–––558.42	<0.001
Permanent Molars (Group III) vs Permanent Molars (Group IV)	42.25	48.77–––525.73	<0.001

**Fig. 1:**
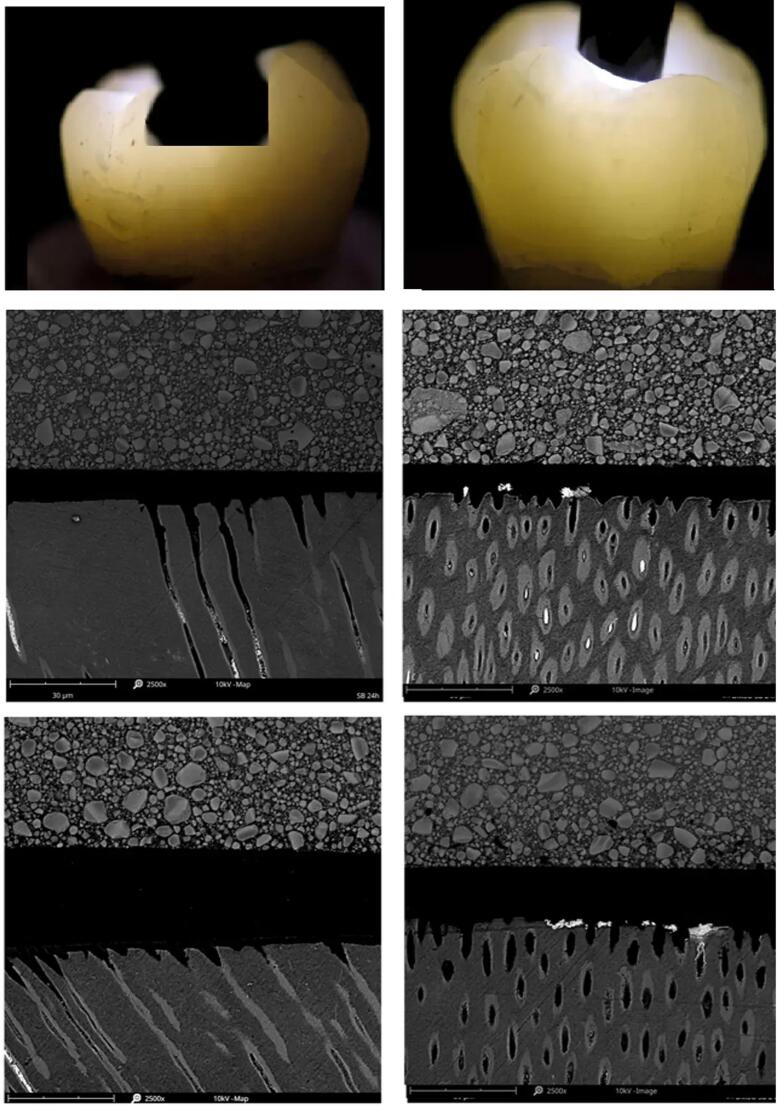

After acquiring high-resolution SEM images of the fracture surfaces, detailed images with magnifications of tens to hundreds of thousands of times were obtained, allowing for the visualisation of fine microstructural details. All irregularities, discontinuities or features that deviated from the surrounding material were spotted and examined. Visible narrow, elongated features with distinct boundaries within the fracture surface were identified and measured in terms of length, orientation and branching patterns. Voids within the fracture surface appeared as empty spaces or regions with lower contrast compared to the surrounding material. Porosity manifested as clusters of small voids or irregularities in the material matrix, while discontinuities or separation layers indicated potential delamination or interfacial debonding between layers of the material or at the interface between the restoration and tooth structure. Any evidence of plastic flow, stretching or tearing was noted, with deformation potentially manifesting as localised bulging, stretching or irregularities in the material morphology (Fig 1.).

The quantitative analysis performed using FEA to characterise the severity and extent of microstructural features observed on the fracture surfaces revealed notable differences between restored deciduous and permanent molars in various mechanical parameters and their associated hazard ratios. Deciduous molars generally exhibited lower values across several parameters compared to permanent molars. For instance, the maximum von Mises stress in deciduous molars ranged from 0.71 to 0.924, while in restored permanent molars, it ranged from 1.326 to 1.908. Similarly, the maximum deformation in deciduous molars ranged from 0.025 to 0.041, whereas in permanent molars, it ranged from 0.0153 to 0.0219. Stress concentration was also lower in deciduous molars, with hazard ratios ranging from 0.63 to 0.84, compared to the higher hazard ratios of 1.302 to 1.416 observed in permanent molars. Deciduous molars exhibited a mixed fracture pattern and fibre pull-out as predominant microstructural features, with hazard ratios ranging from 0.65 to 0.84. By contrast, permanent molars showed an adhesive fracture pattern and crack propagation as the predominant microstructural features, with hazard ratios ranging from 1.02 to 1.04. Mechanical stresses, such as shear stress, compressive stress and tensile stress, tended to be lower in deciduous molars than in permanent molars. The hazard ratios for these parameters in deciduous molars ranged from 0.52 to 0.672, while in permanent molars, they ranged from 1.02 to 1.372 (Supplementary Table 2).

Accordingly, we developed a computational tool to predict the likelihood of fractures in dental composite resin (with a user-friendly web application). The algorithm considers the stress concentration and compares this with the material’s inherent properties to forecast whether a restoration is likely to fail under given conditions. Specific selections may then be made, such as choosing a particular resin material (e.g. nano-filled composites or fibre-reinforced resins), are enabled. The algorithm/tool differentiates between the thickness and remaining (undermined) tooth structure of primary and permanent dentition restorations, along with other geometric factors that significantly influence stress distribution (Supplementary Document).

## 4 Discussion

The fracture resistance concerns the capacity of a material to withstand compressive loads, as this directly impacts its suitability for masticatory load-bearing areas (Al-Nahedh and Alawami, 2020, Battancs et al., 2022, de Abreu et al., 2023). Because of remaining concerns regarding polymerisation kinetics (Al Sunbul et al., 2016, Fronza et al., 2015, Kim et al., 2015), the selection of suitable materials capable of compensating for lost tooth structure is important for improving treatment outcomes. This study compared ultra-polyethylene fibre ribbon, fibre-reinforced composites, such as EverX posterior and EverX flow, and bulk-fill posterior restorative material.

The performance of a restorative material depends on proper isolation and moisture management during the restoration process, ageing of the chosen resin material, its ability to withstand occlusion forces, its resistance to cyclic loading, its durability following cavity preparation and optimal marginal design and its biocompatibility and restoration longevity, as well as any morphological-biological differences between primary and permanent dentitions, including mineral content, tubule densities, and hardness levels (Fronza et al., 2015, Garlapati et al., 2017, Lee et al., 2019, Van Ende et al., 2013). The cavity configuration (C-factor), tooth size, shape, restoration thickness and surface area have also been found to influence the restoration outcomes (Bajunaid et al., 2021, De Oliveira et al., 2020, Tang et al., 2023). Proposing an experimental algorithm aids in meeting the performance requirements that you are listed.

**Fig. 2:**
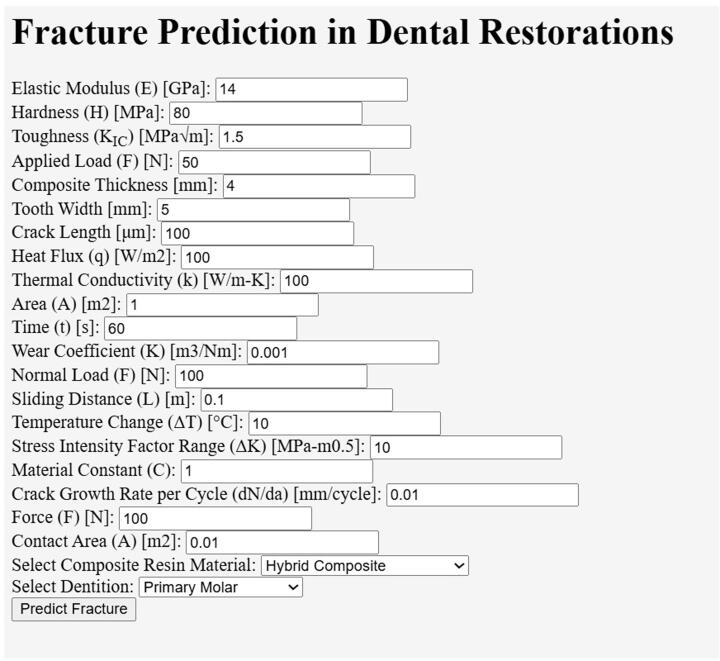

The tool shows interoperability with dental materials science and studies on the effects of nanoparticle additives to predict clinical scenarios (Fig 2.). In dental biomechanics, stress modelling may gauge the gnathic function and parafunction, potentially aiding in the management of aggravated exposure of dental restorations to overload, as occurs in bruxism.

## 5 Conclusion

The study has revealed significant differences in the load-bearing capacity between deciduous teeth and permanent molars, with the latter generally showing higher average maximum compressive loads. High-resolution SEM images showed that there were mainly mixed fracture patterns and fibre breakouts in restored deciduous teeth, while the restored permanent molars showed adhesive fractures and crack propagation. These results, supported by a quantitative analysis using FEA, demonstrate the mechanical behaviour of dental restorations in different tooth types and developmental stages. An understanding of the differences in the resilience of primary teeth and permanent molars enables tailored treatment approaches that ensure dental restorations are optimised based on factors such as tooth type and developmental stage to improve longevity and patient satisfaction.

Nonetheless, it must be noted that the ex-vivo design used in this study limits the capacity to generalise the results to the clinical setting, as the complex oral environment and variations in occlusal forces or patient characteristics were not fully replicated. In addition, the use of specific materials and techniques may focus only on the mechanical aspects of composite resins and neglect possible biological tooth–resin interactions. Furthermore, pH fluctuations, enzymatic activity, and biofilm effects were not studied in this case.

**Funding sources**

No external funding received.

**Conflicts of Interest**: None.

**Authors’ contributions:** Conceptualization: HMHN, DYE, RROOT, SB; Methodology: HMHN, DYE, RROOT, SB; Investigation: HMHN, DYE, RROOT, SB; Data Curation: HMHN, DYE, RROOT, SB; Writing - Original Draft: HMHN, DYE, RROOT, SB; Writing - Review & Editing: HMHN, DYE, RROOT, SB; Visualization: HMHN, DYE, RROOT, SB.

**Institutional Review Board Approval**: Obtained (U6O REC, 2023-7-20).

## Declaration of Competing Interest

The authors declare that they have no known competing financial interests or personal relationships that could have appeared to influence the work reported in this paper.
