# Supplementary material for: Fracture Resistance in Fibre-Reinforced Resin Composite Restorations in Deciduous and Permanent Molars: An Ex Vivo Study
Source: Saudi Dent J. 2024 Jun 12;36(9):1197–202. doi: 10.1016/j.sdentj.2024.06.017 (PMC11402000; doi:10.1016/j.sdentj.2024.06.017)
Supplement: Supplementary Data 1 [file mmc1.docx]

**Supplementary Table 1.** **Materials’ specification, and composition**

| **Materials** | **Composition** |
| --- | --- |
| Polyethylene fibre  (Ribbond-Ultra) | Ultra-high molecular weight polyethylene, Homopolymer H- (CH2-CH2)n-H |
| Fibre-reinforced bulk fill resin composite  (EverX Posterior) | **Organic content** Bisphenol A-glycidyldimethacryla, triethylene glycol dimethacrylate, polymethyl methacrylate. **Inorganic content** E-glass fibres, barium borosilicate glass filler.  **Filler load (wt, vol %)** 74.2/53.6 |
| Flowable fibre-reinforced resin composite  (EverX Flow™) | **Organic content** Bisphenol-A-glycidyl dimethacrylate, tri ethylene glycol dimethacrylate, urethane dimethacrylate,  **Inorganic content**  micrometer-scale E-glass fibre filler, Barium glass  **Filler load (wt, vol %)** 70% by weight, 46% by volume |
| Flowable Bulk Fill resin composite (Tetric N-flow) | **Monomer matrix** Urethane dimethacrylate, Bis-GMA 27.8% Triethyleneglycol dimethacrylate  **Inorganic fillers** - 7.3 Barium glass, ytterbium trifluoride, mixed oxide, silicon dioxide 63.8%.  Additives, stabilizers, catalysts, pigments 1.1% |
| Adhesive  (OptiBond™ Extra Universal) | GPDM Monomer.  Ternary Solvent System (Water, Acetone, Ethanol).  acidity drop. |
| Enamel and dentine etchant  (Meta Biomed) | Phosphoric acid (37%) -H2O -Xanthan gum |
