# Supplementary material for: Fracture Resistance in Fibre-Reinforced Resin Composite Restorations in Deciduous and Permanent Molars: An Ex Vivo Study
Source: Saudi Dent J. 2024 Jun 12;36(9):1197–202. doi: 10.1016/j.sdentj.2024.06.017 (PMC11402000; doi:10.1016/j.sdentj.2024.06.017)
Supplement: Supplementary Data 2 [file mmc2.docx]

Supplementary Table 2

| Parameter | Deciduous Molars | HR (0.65-0.84) | Permanent Molars | Hazzard ratio |
| --- | --- | --- | --- | --- |
| Maximum Von Mises Stress (MPa) | 110 | 0.71- 0.924 | 130 | 1.326-1.908 |
| Maximum Deformation (mm) | 0.025 | 0.025-0.041 | 0.015 | 0.0153-0.0219 |
| Stress Concentration | Moderate | 0.63-0.84 | High | 1.302-1.416 |
| Fracture Pattern | Mixed | 0.65-0.84 | Adhesive | 1.02-1.04 |
| Microstructural Features | Fibre pull-out | 0.65-0.84 | Crack propagation | 1.302-1.416 |
| Shear Stress (MPa) | 70 | 0.52-0.672 | 85 | 1.02-1.04 |
| Compressive Stress (MPa) | 90 | 0.52-0.672 | 110 | 1.02-1.04 |
| Tensile Stress (MPa) | 80 | 0.52-0.672 | 95 | 1.069-1.372 |
| Strain Energy Density (J/m³) | 0.4 | 0.26-0.336 | 0.6 | 0.612-0.876 |
| Contact Pressure (kPa) | 120 | 0.78-0.812 | 140 | 1.928-2.044 |
