# Supplementary material for: Fracture Resistance in Fibre-Reinforced Resin Composite Restorations in Deciduous and Permanent Molars: An Ex Vivo Study
Source: Saudi Dent J. 2024 Jun 12;36(9):1197–202. doi: 10.1016/j.sdentj.2024.06.017 (PMC11402000; doi:10.1016/j.sdentj.2024.06.017)
Supplement: Supplementary Data 3 [file mmc3.docx]

Supplementary Table 3: PICO(T) attributes

| Criterion | Definition | |
| --- | --- | --- |
| Population (P) | Caries-free human mandibular molars (32 deciduous and 32 permanent molars) with deep and wide class I cavities |  |
| Intervention (I) | Four different restorative materials in both dentitions |  |
|  |  | Ribbond-Ultra (polyethylene fibre) + Tetric N Flow (bulk-fill composite) |
|  |  | EverX Posterior (fibre-reinforced bulk fill resin composite) |
|  |  | EverX Flow (fibre-reinforced bulk fill flowable resin composite) |
|  |  | Tetric N Flow (bulk fill composite) |
| Comparison (C) | The four different restorative materials are compared against each other in both dentitions. |  |
| Outcome (O) | Fracture resistance and failure modes, assessed through load-to-fracture tests, light microscopy, scanning electron microscopy (SEM), and finite element analysis (FEA) |  |
| Time (T) | 24 hours in distilled water at 37°C, followed by thermocycling for 2000 cycles (equivalent to six months of oral exposure). |  |
