# Supplementary material for: Fracture Resistance in Fibre-Reinforced Resin Composite Restorations in Deciduous and Permanent Molars: An Ex Vivo Study
Source: Saudi Dent J. 2024 Jun 12;36(9):1197–202. doi: 10.1016/j.sdentj.2024.06.017 (PMC11402000; doi:10.1016/j.sdentj.2024.06.017)
Supplement: Supplementary Data 4 [file mmc4.pdf]

Algorithm Description

To predict the likelihood of fracture in dental restorations, the algorithm requires users to input specific parameters clustered into categories for easier understanding and organization. These categories include material properties, mechanical loads, geometric dimensions, thermal properties, wear and fatigue characteristics, and material and dentition selection. By inputting these values, users can obtain predictions about the stress concentration and the potential for fractures in dental restorations, making the process more streamlined and comprehensive.

Material properties encompass the elastic modulus, hardness, and toughness, which measure the stiffness, resistance to deformation, and fracture resistance of the dental material, respectively. Mechanical loads include the applied load, normal load, and general force applied during the restoration's usage. Geometric dimensions cover the composite thickness, tooth width, crack length, and contact area, which influence the distribution and concentration of stress within the material. Thermal properties, such as heat flux, thermal conductivity, and temperature change, affect the material's response to thermal stress. Wear and fatigue parameters include the wear coefficient, sliding distance, stress intensity factor range, crack growth rate, material constant, and time, all of which impact the material's durability and fatigue resistance. Lastly, material and dentition selection allow users to specify the type of composite resin and dentition, which further refines the predictive accuracy of the algorithm.

Users input these clustered parameters into the algorithm's form, which then calculates the stress concentration factor adjusted for the type of composite resin and dentition. The stress concentration is determined by multiplying this factor by the applied load divided by the product of tooth width and composite thickness. The critical stress is calculated using the toughness divided by the square root of π times the crack length. The algorithm compares the stress concentration to the critical stress to predict whether a fracture is likely to occur. If the stress concentration exceeds the critical stress, a fracture is likely; otherwise, it is less likely. The results, displayed to the user, provide detailed calculations and the likelihood of fracture, helping dental professionals make informed decisions about material selection and design to enhance the durability and reliability of dental restorations.

Material-Specific Parameters

Composite resins have unique properties such as elastic modulus, hardness, and toughness that differ significantly from those of glass ionomers and amalgam. The algorithm uses specific values for these properties to calculate the stress concentration and critical stress accurately. For example:

Elastic Modulus (E) [GPa]: Composite resins have a different stiffness compared to glass ionomers and amalgam.

Hardness (H) [MPa]: Composite resins typically have different hardness values, impacting their resistance to deformation.

Toughness (K_{IC}) [MPa√m]: The fracture resistance of composite resins is distinct from other materials, influencing the critical stress calculation.

Mechanical Load Considerations

Composite resin restorations interact with applied loads differently compared to glass ionomers and amalgam. The algorithm incorporates this by adjusting the stress concentration factor based on the type of composite resin and the dentition type. For instance:

Applied Load (F) [N]: The response of composite resins to applied loads is modeled specifically to account for their behavior, which is different from the more brittle nature of glass ionomers and the metal-based properties of amalgam.

Stress Concentration Factor: This factor is tailored for composite resins, adjusting calculations to reflect the material's ability to distribute stress.

Wear and Fatigue Characteristics

Composite resins wear differently and have distinct fatigue properties compared to glass ionomers and amalgam. The algorithm includes parameters specific to the wear and fatigue of composite resins:

Wear Coefficient (K) [m^3/Nm]: This parameter is unique to the material's wear rate under mechanical loads.

Crack Growth Rate per Cycle (dN/da) [mm/cycle]: Composite resins have specific crack growth rates that differ from those of glass ionomers and amalgam, affecting long-term durability predictions.

Thermal Properties

The thermal response of composite resins is different from that of glass ionomers and amalgam, requiring specific thermal properties to be considered in the algorithm:

Thermal Conductivity (k) [W/m-K]: Composite resins have distinct thermal conductivity, influencing how they handle heat flux and temperature changes.

Temperature Change (ΔT) [°C]: The impact of temperature changes on composite resins is modeled to reflect their unique expansion and contraction behavior.

Material Constant (C): Specific to the composite resin, influencing fatigue calculations.

Composite Resin Material Type: Allows the user to select the specific type of composite resin (e.g., hybrid, microfilled), which adjusts the stress concentration factor and other parameters accordingly.

Exclusion of Other Materials

The properties, mechanical load considerations, wear and fatigue characteristics, and thermal properties for glass ionomers and amalgam are not included in the algorithm. This exclusion ensures that the predictions are accurate for composite resins but would not be applicable to other materials, highlighting the customization for composite resin restorations specifically. By focusing on the unique properties and behavior of composite resins, the algorithm provides accurate and reliable predictions for fracture likelihood in dental restorations made from these materials, ensuring that dental professionals can make informed decisions tailored to the specific material in use.

Complementary Use in Restorative Procedures

This algorithm for predicting fracture in dental restorations, specifically tailored for composite resins, is not directly interoperable with endodontics. Endodontics primarily deals with the treatment of the dental pulp and root canal system, focusing on issues like infection, inflammation, and root canal therapy. However, there are areas where the algorithm could potentially interact with or complement endodontic treatments. After a root canal treatment, a tooth often requires a restoration to ensure structural integrity and function. Composite resins are commonly used for such restorations. The algorithm could help in predicting the likelihood of fracture in these composite restorations, which are placed after endodontic therapy. Ensuring the durability of these restorations is critical, especially since endodontically treated teeth are often more brittle and prone to fracture. The algorithm can assist in selecting the appropriate composite resin material for restoring endodontically treated teeth. By inputting the specific parameters and conditions of the treated tooth, the algorithm can predict the most suitable composite resin type that would minimize the risk of fracture.

Endodontically treated teeth may experience different stress distributions due to changes in their structure and material properties after the removal of pulp tissue. The algorithm can be used to analyze stress concentrations in composite restorations on these treated teeth, aiding in planning and executing more durable restorative procedures. Teeth that have undergone endodontic treatment are more susceptible to crack propagation. By using the algorithm, dental professionals can monitor and predict the potential for crack growth in composite restorations placed on endodontically treated teeth, helping to take preemptive measures to reinforce the restoration.

For the algorithm to be fully interoperable with endodontics, it would need to incorporate endodontic-specific factors such as the impact of different root canal filling materials, the presence of posts, and the condition of the remaining tooth structure after endodontic therapy.

The current form focuses on the properties of composite resin restorations. To be truly interoperable with endodontics, it would need modifications to account for the altered biomechanical properties of endodontically treated teeth, which are typically more fragile and have different stress distribution characteristics.

By integrating the algorithm into a comprehensive treatment planning system, dental professionals can use it alongside endodontic treatment protocols. This integration would involve using the algorithm to predict and optimize the outcomes of restorative treatments following endodontic procedures, ensuring the long-term success of the treated teeth. The algorithm can serve as an educational tool for endodontists and restorative dentists, helping them understand the interplay between endodontic treatments and subsequent restorative procedures. This understanding can lead to better-informed decisions regarding material selection and restoration techniques.

For full interoperability, additional customization and integration of endodontic-specific factors would be necessary.

Limitations

Future endeavours should assess:

Structural Integrity Post-Endodontics:

Remaining Tooth Structure (RTS) [%]: Percentage of original tooth structure remaining after endodontic treatment.

Presence of Posts (yes/no): Whether a post is used to reinforce the tooth structure.

Type of Post (fiber, metal, ceramic): The material used for the post, affecting the stress distribution.

Root Canal Filling Material Properties:

Filling Material Type (gutta-percha, resilon): Different materials used for filling the root canal, which have varying mechanical properties.

Adhesive Strength [MPa]: The bond strength of the filling material to the dentin.

Endodontic Treatment Details:

Number of Canals Treated: The number of root canals treated, affecting the internal structure.

Condition of Apical Seal (good, moderate, poor): Quality of the seal at the tip of the root canal.

Stress Distribution Adjustments:

Adjust the stress concentration factor based on the remaining tooth structure (RTS). Endodontically treated teeth with less remaining structure are more susceptible to fractures.

Incorporate the type and presence of posts into the stress distribution calculations, as posts help to distribute stress more evenly and reduce the likelihood of fractures.

Material Interaction Effects:

Consider the interaction between the composite resin restoration and the root canal filling material. Different materials expand and contract differently under thermal and mechanical loads, influencing stress concentrations.

Calculate the adhesive strength between the composite resin and the root canal filling material to predict potential points of failure.
